# The impact of BMI on sperm parameters and the metabolite changes of seminal plasma concomitantly

**DOI:** 10.18632/oncotarget.14950

**Published:** 2017-02-01

**Authors:** Dan Guo, Wei Wu, Qiuqin Tang, Shanlei Qiao, Yiqiu Chen, Minjian Chen, Mengying Teng, Chuncheng Lu, Hongjuan Ding, Yankai Xia, Lingqing Hu, Daozhen Chen, Jiahao Sha, Xinru Wang

**Affiliations:** ^1^ State Key Laboratory of Reproductive Medicine, Institute of Toxicology, School of Public Health, Nanjing Medical University, Nanjing, China; ^2^ Key Laboratory of Modern Toxicology of Ministry of Education, Nanjing Medical University, Nanjing, China; ^3^ State Key Laboratory of Reproductive Medicine, Wuxi Maternal and Child Health Care Hospital Affiliated to Nanjing Medical University, Wuxi, China; ^4^ Department of Obstetrics, State Key Laboratory of Reproductive Medicine, Obstetrics and Gynecology Hospital Affiliated to Nanjing Medical University, Nanjing, China; ^5^ State Key Laboratory of Reproductive Medicine, Nanjing Medical University, Nanjing, China

**Keywords:** BMI, obesity, sperm parameters, meta-analysis, metabolomic analysis, Pathology Section

## Abstract

The development of male infertility increased rapidly worldwide, which coinciding with the epidemic of obesity. However, the impact of weight abnormalities on sperm quality is still contestable. To assess the correlation between BMI and sperm parameters, we searched relevant articles in PubMed, Embase, Web of science, and Wanfang database published until June 2015 without language restriction. Otherwise, we also recruited some participants who attended fertility clinic as well as some general populations in this report. We performed a systematic review and meta-analysis about BMI and sperm parameters containing total sperm count, concentration, semen volume and sperm motility (overall and progressive). Metabolomic analysis of seminal plasma was performed to explore the mechanism from a new perspective. This study found standardized weighted mean differences (SMD) in sperm parameters (total sperm count, sperm concentration, and semen volume) of abnormal weight groups decreased to different degree compared to normal weight. Dose-response analysis found SMD of sperm count, sperm concentration and semen volume respectively fell 2.4%, 1.3% and 2.0% compared with normal weight for every 5-unit increase in BMI. Metabolomic analysis of seminal plasma showed that spermidine and spermine were likely to play a vital role in the spermatogenesis progress. This systematic review with meta-analysis has confirmed there was a relationship between BMI and sperm quality, suggesting obesity may be a detrimental factor of male infertility.

## INTRODUCTION

The development of assisted reproductive technology and its exponential increase usage have reflected, to a certain extent, infertility has become a serious worldwide problem. Forty-eight point five million couples were infertile in 2010, and about 50% infertility is caused by male factors [1, 2]. Sperm quality and spermatogenesis is vital for male fertility. The stand or fall of these situations depends on multi-factors i.e. genetic, environmental, behavioral or dietary. As a growing social health problem, the effect of obesity is not to be sneezed at that costs to both the community and the individual since obesity may be related to cardiovascular disease, diabetes and cancers [3, 4]. Along with the prevalence of obesity in the world that the proportion of men who are overweight (BMI ≧ 25) is 36.9% in 2013 [5], and at the same time, infertility often coexists with obesity, many investigators put their eyes on the impact of body mass index on sperm quality. Since the beginning of this century, relevant researches emerged in endlessly. However, there is still a debate about whether overweight/obesity is a risk factor for infertility. Shayeb et al (2011) and Duits et al (2010) found high BMI mainly caused low semen volume and had no effect on other sperm parameters [6, 7]. Stephanie Belloc et al concluded increased BMI affected sperm quality including sperm count, concentration, volume and motility [8]. However, Aggerholm et al (2008) got the conclusion that there was no statistically significant relationship between BMI and sperm count and concentration [9]. Two meta-analyses published in 2010 and 2013 revealed different conclusions that MacDonald et al found no relationship between obesity and sperm concentration or sperm count, while Sermondade et al showed obesity increased the risk of abnormal sperm count [10, 11]. Since 2013, many new relevant researches published in succession, including some consistent with the conclusion of MacDonald et al [12–15], and some got the same result with Sermondade et al [8, 16–19]. It was surprising the participant numbers of some studies are unprecedented, for instance, Stephanie Belloc et al recruited 10665 men and Chih-Wei Tsao et al recruited 7630 men to evaluate the association between BMI and semen characteristics in 2014 and 2015, respectively [8, 18].

We summarized these new studies combined with the past as well as our personal data, and conducted this study to investigate the effect of overweight and obesity on several sperm parameters. Besides, because obesity is a metabolic disorder phenomenon, a proper understanding of small molecule metabolites in human seminal plasma will provide biological information on mechanism underlying spermatogenesis. Therefore, we also performed the metabolomic analysis of seminal plasma in this study.

## RESULTS

### Result of search

**Figure 1:**
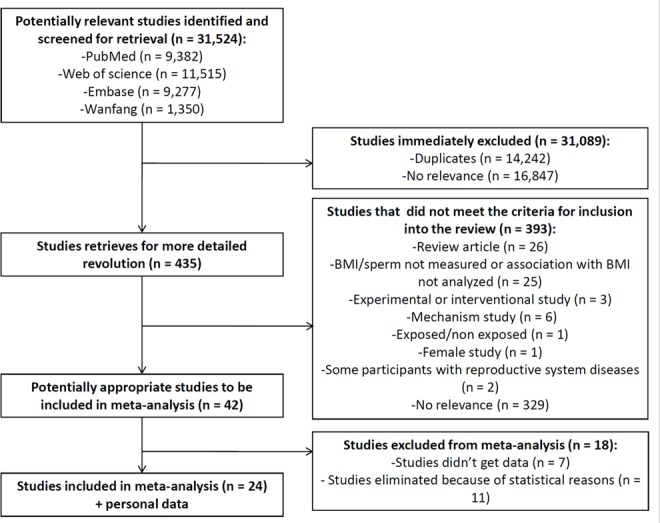
Flow chart of study selection

The search strategy identified a total of 31524 articles, including 9382 from PubMed, 11515 from Web of Science, 9277 from Embase and 1350 from Wanfang; however, 31089 studies were excluded because 14242 were reduplicative and 16847 articles had no relevance to the primary research. We selected 42 articles that providing BMI and sperm parameters data after review of 435 abstracts. Among these, 24 articles studying the relationship between BMI and sperm parameters seemed potentially appropriate to be included in the meta-analysis (Figure 1).

### Description of studies and participants

The present meta-analysis included 25 studies (Tables 1-2) included in total 26814 participants, among those 2106 participants are from our own study. All these were cross-sectional studies except three prospective studies and one retrospective study that reported cross-sectional data as well as a case-control study. Apart from five without mentioned and five self-reported, data of weight and height in other studies were absolutely measured on site by trained personnel. Study participants were recruited from either a general population or the infertility populations from fertility clinic, with one exception of attending physical examination for military service. Sperm analysis was performed followed WHO 1999 guidelines or WHO 2010 guidelines for all studies, except one which is based on the WHO Laboratory Manual for Examination of Human Semen and Sperm-Cervical Mucus Interactions, Cambridge (2001) [19]. Amongst all the studies in this meta-analysis, researched outcomes were as follows: sperm count (19/25), concentration (23/25), volume (20/25), motility (18/25), and progressive motility (12/25). The seven studies [6–9, 20, 21] with the larger sample size (over 1000) accounted for the major part of the meta-analysis and came to several different results as shown above.

**Table 1:** Characteristics of observational studies included in this review (Part 1)

Study	Location	Study group	Ascertainment of BMI	Repeated semen collection	BMI distribution	Total sperm count	Sperm concentration	Semen volume	Sperm motility	Sperm motility (progressive)
						Mean ± SD/ Median (IQR)	Mean ± SD/ Median (IQR)	Mean ± SD/ Median (IQR)	Mean ± SD/ Median (IQR)	Mean ± SD/ Median (IQR)
Belloc et al (2014)	France	10170 men referred in the course of a couple infertility evaluation of any origin	Self-reported	once	18.5-24.9 (n = 5799)	171 ± 170	56.4 ± 54.9	3.3 ± 1.6	39.7 ± 16.7	36.9 ± 16.8
					25.0-29.9 (n = 3607)	163 ± 175	55.1 ± 56.9	3.2 ± 1.5	39.4 ± 16.6	36.5 ± 16.8
					30.0-34.9 (n = 634)	141 ± 166	50.7 ± 55.7	3.1 ± 1.6	37.5 ± 16.6	34.4 ± 16.9
					35.0-39.9 (n = 97)	136 ± 144	49.7 ± 49.5	3.0 ± 1.8	38.5 ± 15.8	35.6 ± 15.9
					≥40.0 (n = 33)	92 ± 95	39.4 ± 51.0	2.7 ± 1.6	38.0 ± 16.2	34.7 ± 17.1
Guo et al (unpublished)	China	2106 men including 645 fertile men and 1461 infertile men from maternity clinic and infertility clinic respectively	Self-reported	once	18.5-24.0 (n = 1057)	157.94 ± 201.33	49.90 ± 43.60	3.18 ± 2.32	46.98 ± 23.86	37.76 ± 20.59
					24.1-27.9 (n = 798)	154.32 ± 165.89	50.90 ± 45.73	3.07 ± 1.52	47.70 ± 23.37	39.06 ± 20.33
					≥28.0 (n = 251)	141.26 ± 143.80	49.06 ± 39.53	2.91 ± 1.43	47.97 ± 23.83	39.14 ± 21.22
Paasch et al (2010)	Germany	2058 patients aged 17-67 years attending the clinic examining the factors affecting semen quality excluding reproductive related diseases and chronic diseases	NA	once	20-25 (n = 1003)	159.2 ± 5.51a			40.9 ± 1.01a	
					25-30 (n = 810)	143.7 ± 6.26a			39.7 ± 0.99a	
					≥30 (n = 245)	143.0 ± 11.68a			38.1 ± 2.06a	
Shayeb et al (2011)	United Kingdom	2017 male partners of couples attending for infertility investigations at the Aberdeen Fertility Clinic from 1990 to 2007	NA	once	18.5-24.99 (n = 839)	144.0 (61.1-290.8)c	47.9 (22.0-84.3)c	3.5 ± 1.8	45.0 (29.4-59.0)c	
					25.0-29.99 (n = 909)	153.0 (58.8-273.4)c	47.0 (21.0-82.0)c	3.5 ± 1.8	45.4 (27.6-59.0)c	
					≥30 (n = 269)	162.7 (48.9-259.8)c	50.8 (21.3-83.0)c	3.2 ± 1.7	47.0 (27.0-61.0)c	
Aggerholm et al (2008)	Denmark	1922 men without prior knowledge about their fertility aged 18-66 from five separate occupational or environmental semen studies	Self-reported	once	20.0-25.0 (n = 986)	161 (77-309)	55 (9-99)			40 (19-66)
					25.1-30.0 (n = 773)	153 (67-286)	53 (27-90)			52 (28-66)
					>30.0 (n = 163)	156 (75-317)	65 (33-114)			59 (40-72)
Jensen et al (2004)	Denmark	1341 all 18-year-old men attending a compulsory physical examination for military service excluding chronic diseases	Measured on site	once	20-25 (n = 1042)	138.0 (59-259)	46.0 (23-84)	3.2 ± 1.4	65.4 ± 12.4	
					≥25 (n = 299)	116.0 (46-213)	39.0 (20-69)	3.2 ± 1.6	65.5 ± 12.5	
Duits et al (2010)	the Netherlands	1366 men visiting the Centre for Reproductive Medicine as part of a subfertile couple from 2000 to 2007	Self-reported	at least two semen analyses	20.1-25 (n = 633)	174.3 (50.2-219.6)	53.3 (18.0-89.0)	3.7 ± 2.5		31.1 ± 15.9
					25.1-30 (n = 587)	153 (48.8-283.7)	56 (17.8-96.8)	3.3 ± 1.7		32.5 ± 15.9
					>30 (n = 146)	135.6 (45.8-261.6)	47 (17.4-86.5)	3.4 ± 1.6		33.5 ± 16.2
Xiao et al (2013)	China	786 men attending the reproductive center infertility clinic in 2010-2013	Measured on site	once	18.5-23.9 (n = 401)	166.5 (70.4-298)	71 (27-111)	2.5 (2-3.5)		48 (28.0-60.0)
					24-27.9 (n = 221)	126 (64.6-215)	70 (32-98.5)	2 (1.5-3)		48.0 (31.5-59.5)
					28-29.9 (n = 120)	121 (62.3-215.1)	63 (28-103)	2 (1.3-2.8)		45 (35.0-57.0)
					≥30 (n = 44)	125 (46.1-288.8)	64.5 (37.3-112.3)	2 (1.3-2.5)		44.5 (33.3-54.5)
Martini et al (2010)	Argentina	794 male partner of couples attending the Andrology and Reproduction Laboratory in Cordoba, Argentina in 2006-2007	Measured on site	once	18.5-24.9 (n = 251)		43.7 ± 1.9a	3.2 ± 0.1a	51.4 ± 1.2a	39.8 ± 1.2a
					25.0-29.9 (n = 388)		44.2 ± 1.8a	3.1 ± 0.1a	50.2 ± 1.0a	38.8 ± 0.9a
					30-50 (n = 155)		43 ± 3.2a	3.1 ± 0.1a	46.6 ± 1.7a	35.9 ± 1.6a
Eisenberg et al (2014)	USA	468 men of couples attempting to conceive in two geographic areas (Texas and Michigan, USA) from the LIFE study in 2005-2009	Measured on site	once or twice	<25 (n = 83)	198.5 (112.8-336.9)	55.3 (34.4-94.1)	3.5 (2.4-4.8)	70.7 (64.8-75.5)	
					25-30 (n = 191)	190.6 (100.3-338.1)	63.2 (38.0-92.5)	3.4 (2.1-4.7)	67.6 (60.1-73.4)	
					30-35 (n = 122)	186.5 (99.1-305.1)	62.4 (31.9-100.4)	3.2 (2.3-4.1)	66.6 (60.3-73.2)	
					≥35 (n = 72)	141.7 (58.4-286.5)	60.0 (25.5-100.4)	2.8 (1.8-3.9)	70.2 (61.7-75.9)	
Macdonald et al (2012)	New Zealand	511 men attending the fertility clinic for semen analysis or therapeutic procedures at three fertility clinics in Auckland, New Zealand in 2008-2012	Measured on site 85%, self-reported 15%	once	18.5-24.99 (n = 139)	128.1 (21.1-413)d	52.5 (7.4-139) d	2.8 1.3-5.2) d	64.0 (35.0-80.0)d	
					25-29.99 (n = 253)	135.0 (24.1-455)d	50.0 (9.3-152)d	3.0 (1.5-5.2)d	64.5 (40.0-81.0)d	
					≥30 (n = 119)	122.1 (15.4-407)d	42.0 (7.4-116.5)d	2.9 (1.4-5.2)d	67.0 (44.0-82.0)d	
Chavarro et al (2010)	MA	483 male partners in subfertile couples presented for evaluation at the MGH Fertility Center in 2000-2006	Measured on site	once	18.5-24.9 (n = 123)	257 (102-477)	76 (35-155)	3.2 (2.2-4.2)	49 (30 -70)	
					25-29.9 (n = 233)	229 (87-414)	81 (32-172)	2.9 (1.9-4.1)	55 (35-69)	
					30-34.9 (n = 87)	204 (92-390)	87 (41-154)	3.0 (1.8-3.5)	54 (30-71)	
					≥35 (n = 40)	167 (78-293)	77 (23-148)	2.6 (1.9-4.0)	55 (25 -68)	

**Table 2:** Characteristics of observational studies included in this review (Part 2)

Study	Location	Study group	Ascertainment of BMI	Repeated semen collection	BMI distribution	Total sperm count	Sperm concentration	Semen volume	Sperm motility	Sperm motility (progressive)
						Mean ± SD/ Median (IQR)	Mean ± SD/ Median (IQR)	Mean ± SD/ Median (IQR)	Mean ± SD/ Median (IQR)	Mean ± SD/ Median (IQR)
Hammiche et al (2012)	The Netherlands	450 men of subfertile couples visiting a tertiary outpatient clinic for reproductive treatment or specialized medical preconception care in 2007-2010	Measured on site	once	<25 (n = 153)	68.6 (19.8-183.2)	34.0 (8.9-62.3)	3.0 (1.9-4.0)		39.0 (22.0-48.5)
					25.0-29.9 (n = 225)	49.6 (14-124.8)	23.0 (6.8-51.5)	2.7 (1.5-3.5)		37.0 (21.0-47.0)
					≥30 (n = 72)	45.9 (2.8-147.5)	18.0 (1.1-60.3)	2.4 (1.6-3.4)		39.0 (23.0-49.0)
Cheng et al (2013)	China	403 men of subfertile couples attending the infertility clinic of Ningxia Medical University General Hospital in 2008-2012	Measured on site	once	18.5-24 (n = 182)		48.36 ± 28.81	2.52 ± 0.61	52.73 ± 22.02	35.82 ± 15.29
					24-28 (n = 154)		54.09 ± 32.92	2.55 ± 0.64	58.71 ± 20.49	39.53 ± 14.74
					28-30 (n = 35)		54.32 ± 31.79	2.62 ± 0.77	61.23 ± 21.60	38.47 ± 16.58
					≥30 (n = 32)		39.04 ± 23.02	2.62 ± 0.78	48.87 ± 23.18	34.81 ± 13.73
Zhu et al (2014)	China	318 infertile men attending the infertility clinic in 2012	Measured on site	once	18.5-23.9 (n = 138)	81.1 ± 59.3	24.7 ± 18.6	3.51 ± 1.4		26.7 ± 15.4
					24-27.9 (n = 116)	56.2 ± 49.7	18.4 ± 16.3	3.22 ± 1.43		24.3 ± 14.9
					≥28 (n = 64)	45.4 ± 41.1	14.8 ± 12.6	3.27 ± 1.87		20.2 ± 14.0
Koloszar et al (2005)	Hungary	245 normozoospermic male patients of reproductive age attending clinic of infertility problems	Measured on site	NA	20.1-25 (n = 96)		39 ± 14			
					25.1-30 (n = 91)		37 ± 14			
					>30 (n = 58)		29 ± 12			
Ehala-Aleksejev et al (2015)	Estonia	260 male partners of pregnant women presenting for prenatal care	Measured on site	NA	<25 (n = 127)	316.9 (275.7-364.6)b	80.5 (69.4-93.3)b	3.9 (3.7-4.2)b	50.2 (48.0-52.4)b	
					25.0-29.9 (n = 95)	260.6 (218.0-311.6)b	66.1 (56.7-77.0)b	3.9 (3.6-4.3)b	52.0 (49.6-54.5)b	
					≥30 (n = 38)	223.6 (164.2-304.5)b	69.4 (52.7-91.4)b	3.2 (2.8-3.8)b	54.2 (50.8-57.5)b	
Bai et al (2014)	China	177 infertile men aged 23-50 from infertility clinic of Second Affiliated Hospital of Kunming Medical University	Measured on site	once	<24 (n = 58)	146.39 ± 111.87	53.09 ± 34.30	2.71 ± 0.92	58.50 ± 24.19	50.60 ± 23.23
					24-27.99 (n = 60)	131.14 ± 94.90	57.88 ± 37.57	2.27 ± 0.98	58.00 ± 23.96	51.70 ± 21.92
					>28 (n = 59)	103.75 ± 78.38	52.44 ± 34.06	1.96 ± 0.7e	54.17 ± 26.06	46.49 ± 25.18
Mormandi et al (2013)	Argentina	168 patients attendeding the andrology section of the endocrinology of Hospital Durand for infertility in 2008-2010	Measured on site	at least once	20-24.9 (n = 34)		37.5 (5.4-119)e	2.8 (0.9-6)e	50 (0-85)e	
					25-29.9 (n = 100)		30 (3.6-325)e	2.5 (0.2-7)e	60.5 (0-90)e	
					≥30 (n = 34)		33.5 (2-268)e	2.3 (0.3-5.2)e	47.5 (10-85)e	
Andersen et al (2015)	Norway	166 men aged 18 years and older from two general population through advertisement and a fertility clinic in 2008-2013	Measured on site	once	18.5-24.9 (n = 45)	205 (7-1862) e	53 (1.3-222)e		63 (17-74)e	
					25-29.9 (n = 52)	190 (7-601)e	60 (3.6-350)e		41 (1-76)e	
					30-34.9 (n = 31)	244 (6-1290)e	54.9 (3.8-305)e		43 (10-70)e	
					≥35 (n = 38)	121 (20-1127)e	41.5 (3.0-281)e		30 (0-43)e	
Hajshafiha et al (2013)	Iran	151 male patients living as a partner in an infertile couple (fertile 83, infertile 68) seeking infertility treatment	Measured on site	twice	20.1-25.0 (n = 66)	115.84 ± 65.1			47.56 ± 18.2	
					25.1-30.0 (n = 66)	116.3 ± 71.6			41.78 ± 19.6	
					>30.0 (n = 19)	115.36 ± 74.8			46.52 ± 18.7	
Rybar et al (2011)	Czech Republic	153 men from couples attending an infertility clinic who had tried for 12 months or more to achieve pregnancy without success	NA	NA	<24.9 (n = 74)		61.0 ± 45.8	3.8 ± 1.6	54.4 ± 11.1	
					25-29.9 (n = 63)		60.5 ± 39.5	3.6 ± 1.6	53.4 ± 9.8	
					>30 (n = 16)		70.8 ± 43.6	4.5 ± 1.8	53.8 ± 12.0	
Vignera et al (2012)	Italy	150 general-based populations containing 50 normal-weight, 50 overweight and 50 obese men selected randomly	Measured on site	twice	19.0-24.9 (n = 50)	211.1 ± 30.2a	66.0 ± 5.3a	3.2 ± 0.6a		48.4 ± 4.4a
					25.1-29.9 (n = 50)	225.1 ± 44.4a	68.2 ± 11.0a	3.3 ± 0.4a		20.2 ± 4.0a
					30.1-44.0 (n = 50)	191.7 ± 26.4a	57.9 ± 9.7a	3.3 ± 0.8a		23.2 ± 6.0a
Gutorova et al (2014)	Russia	99 volunteers aged 23-58 years born in Arkhangelsk or lived there for at least 17 years	Measured on site	once	18.5-24.9 (n = 36)	156.42 ± 27.96aa	51.30 ± 8.48 a	3.36 ± 0.35a	43.75 ± 5.07a	
					25.1-29.9 (n = 44)	215.50 ± 22.95a	67.94 ± 6.94 a	3.24 ± 0.28a	52.10 ± 4.16a	
					≥30.1 (n = 19)	113.35 ± 34.64a	40.20 ± 10.48 a	2.88 ± 0.42a	37.23 ± 6.28a	
Bai et al (2015)	China	26 infertile men aged 21-50 from infertility clinic and 26 healthy control from maternity clinic for the second child	Measured on site	once	18.5-24 (n = 26)		59.58 ± 30.30	2.46 ± 0.94	66.38 ± 14.30	57.54 ± 16.94
					24-28 (n = 12)		62.58 ± 29.30	2.51 ± 0.74	62 ± 14.52	54.67 ± 15.74
					≥28 (n = 14)		67.50 ± 36.76	2.53 ± 1.03	61.57 ± 13.58	53.64 ± 13.78

^a^ Mean±SE; ^b^ mean (95% CI); ^c^ mean (IQR); ^d^ median (10th-90th percentile); ^e^ median (range)

### Personal data result

There was no relationship between BMI and sperm parameters on the whole personal participants (Supplementary Table 1). Compared with normal weight, weight abnormalities had no effect on the low sperm parameters of total people (Supplementary Table 2), and had no effect on the low sperm parameters of fertile parts only (Supplementary Table 3), while obesity was associated with significantly increased ORs for low sperm count (OR = 2.69), sperm concentration (OR = 1.87), sperm motility (OR = 2.47) and sperm progressive motility (OR = 2.03) in infertile men (Supplementary Table 4) (all results were adjusted for participants age).

### Impact of BMI on sperm parameters

**Figure 2:**
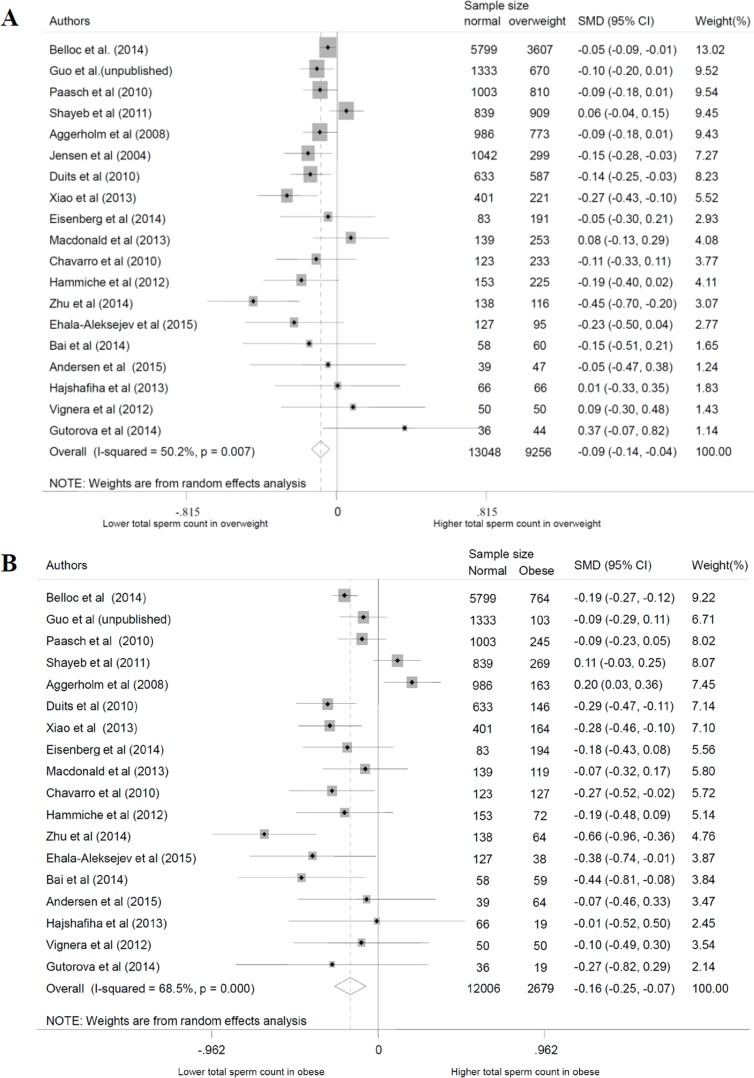
Forest plot of abnormal body mass and total sperm count compared with normal body mass. Each point represents a separate study for the indicated association. A. overweight; B. obese

**Figure 3:**
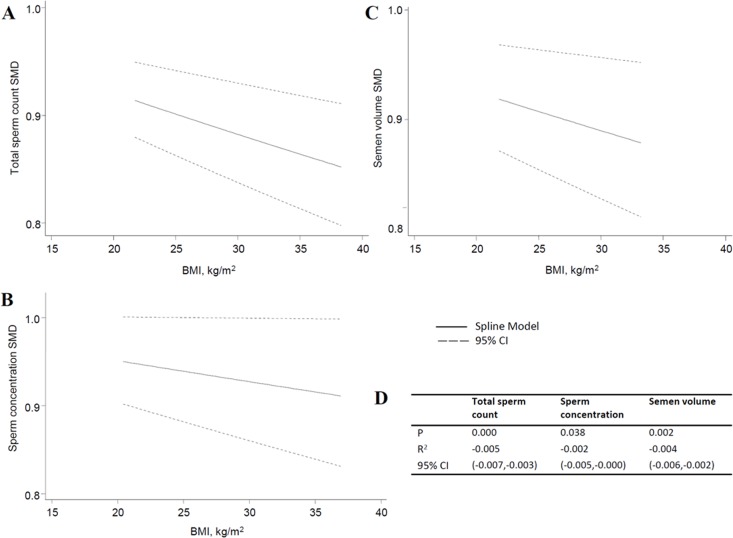
Dose-response of total sperm count, sperm concentration and semen volume. A. total sperm count; B. sperm concentration; C. semen volume; D. the parameters of dose-response

In this review, we used a total of 24 studies and personal data to perform the meta-analysis. With the normal weight participants as the reference group, the standardized weighted mean differences (SMD) of sperm parameters of abnormal weight group was calculated (Table 3, Figure 2 for data of total sperm count). From the meta-analysis, we found overweight decreased the quality of total sperm count and semen volume (P = 0.000 and 0.002), obesity decreased the quality of total sperm count, sperm concentration, and semen volume (P = 0.001, 0.006 and 0.000, respectively), while changes of sperm motility didn't show significant statistical difference, and are not shown on a specific figure. For dose-response analysis, only seven articles had the mean values of the BMI categories that were used in our analysis. The results showed sperm count, sperm concentration and semen volume were weakened with the increase of BMI with the P values were 0.000, 0.038 and 0.003, respectively (Figure 3). And, for every 5-unit increase in BMI, the SMD fell 2.4%, 1.3% and 2.0% compared with normal weight, respectively.

**Table 3:** SMD and 95% CI of sperm parameters in abnormal weight groups

	Overweight		Obese	
	SMD (95% CI)	*P*	SMD (95% CI)	*P*
Total sperm count	−0.093 (−0.142, −0.043)	<0.001	−0.162 (−0.254, −0.070)	0.001
Sperm concentration	−0.038 (−0.084, 0.007)	0.098	−0.118 (−0.202, −0.034)	0.006
Semen volume	−0.095 (−0.156, −0.033)	0.002	−0.182 (−0.275, −0.088)	<0.001
Sperm motility	0.015 (−0.057, 0.086)	0.691	−0.108 (−0.234, 0.018)	0.092
Sperm progressive motility	0.004 (−0.090, 0.097)	0.939	−0.089 (−0.227, 0.050)	0.211

All SMD are relative to normal weight (BMI between 18.5-25.0).

### Sensitivity analyses

**Figure 4:**
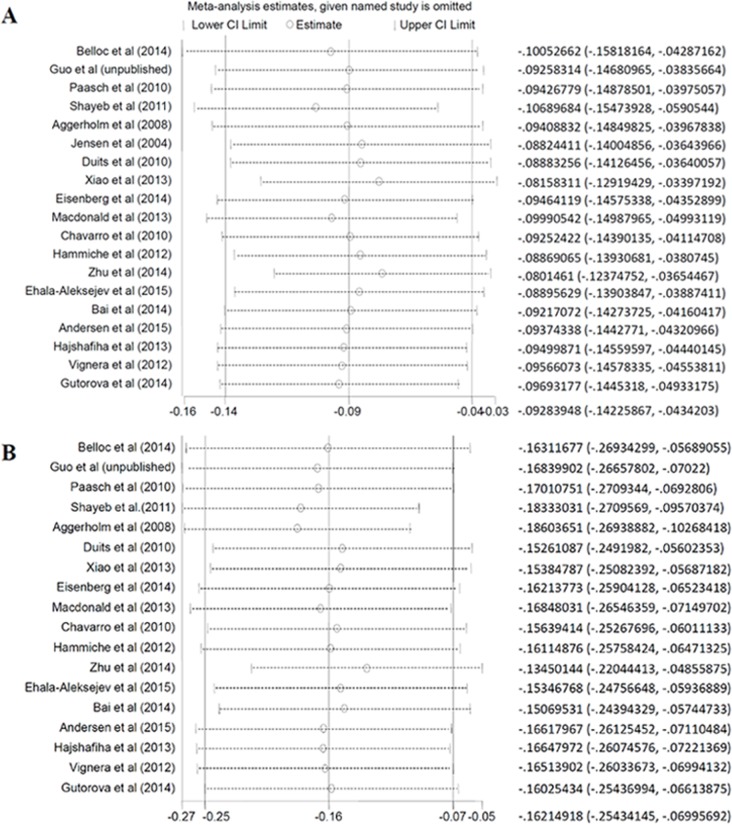
Sensitivity analysis about total sperm count. A. overweight; B. obese

To reduce the heterogeneity between these studies, random effected models were performed in this meta analysis. Then, we conducted the sensitive analyses to access whether modification of the inclusion criteria affected the final results. The results showed that the SMD of progressive motility in obese group changed when eliminated data from Aggerholm et al (2008) [9], while the outcome of other groups was not qualitatively changed with or without any study. Also, there was no obvious influence on the results after taking out of the data from our data or Belloc et al (2014) [8] (Figure 4).

### Subgroup analyses

**Figure 5:**
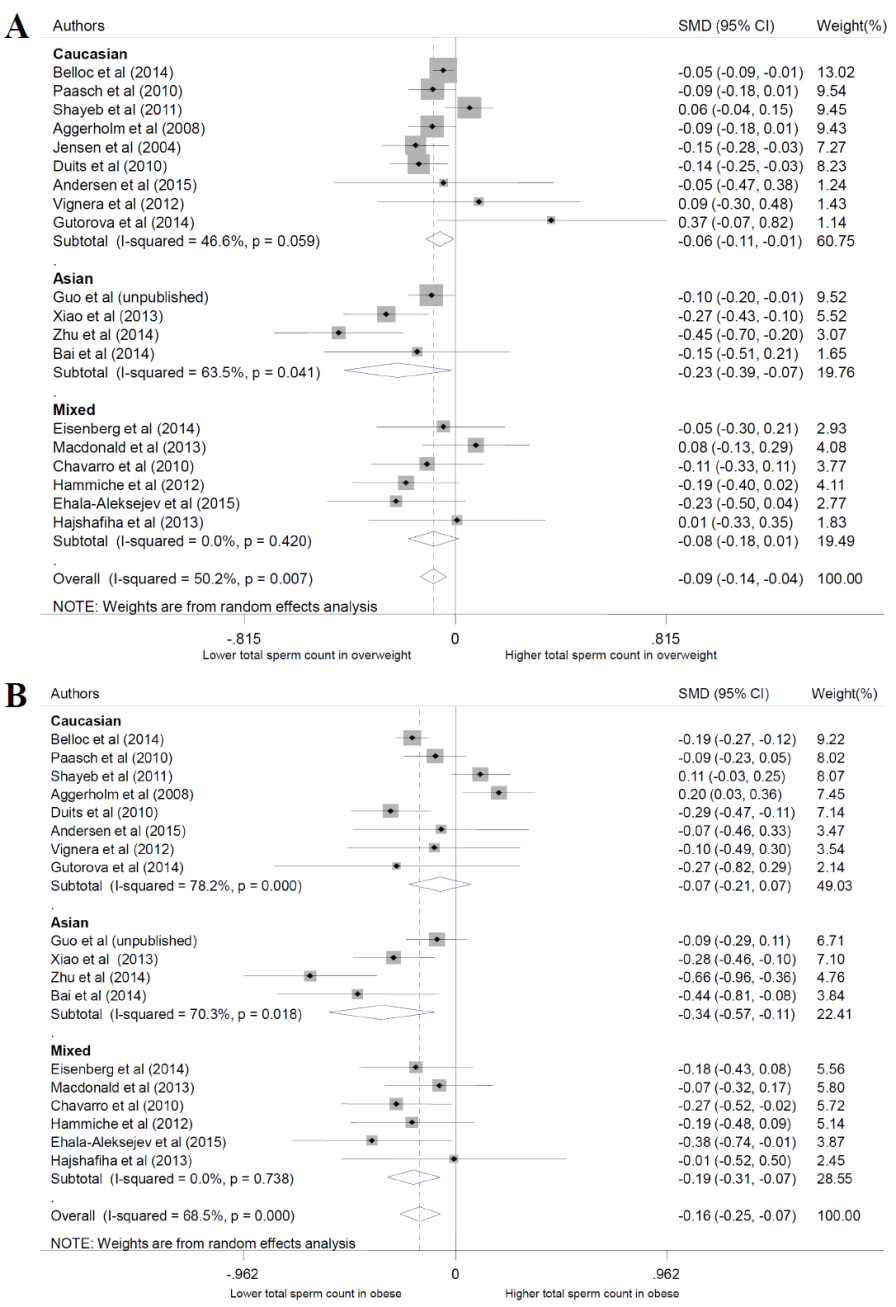
Subgroup analysis based on ethnicity about total sperm count. A. overweight; B. obese

Subgroup analyses were conducted based on the ethnicity of participants. The results showed that the sperm parameters were different in Caucasian and Asian population in some BMI groups, such as total sperm count, semen volume and sperm motility in the obese group, but the effect of BMI was similar (Figure 5).

### Assessment of publication bias

**Figure 6:**
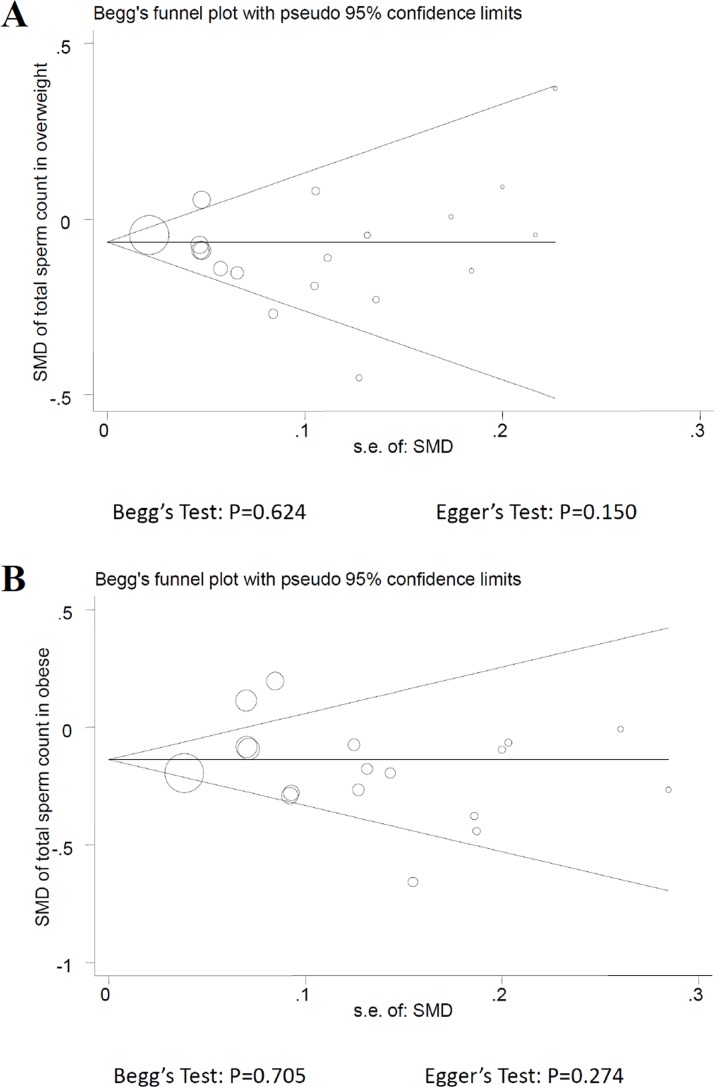
Funnel plot of observational studies about total sperm count. A. overweight; B. obese

Using Egger's test and Begg's funnel plot to evaluate the publication bias of studies, the results provided no evidence in each sperm parameter of different groups (Figure 6).

### Trial sequential analyses

**Figure 7:**
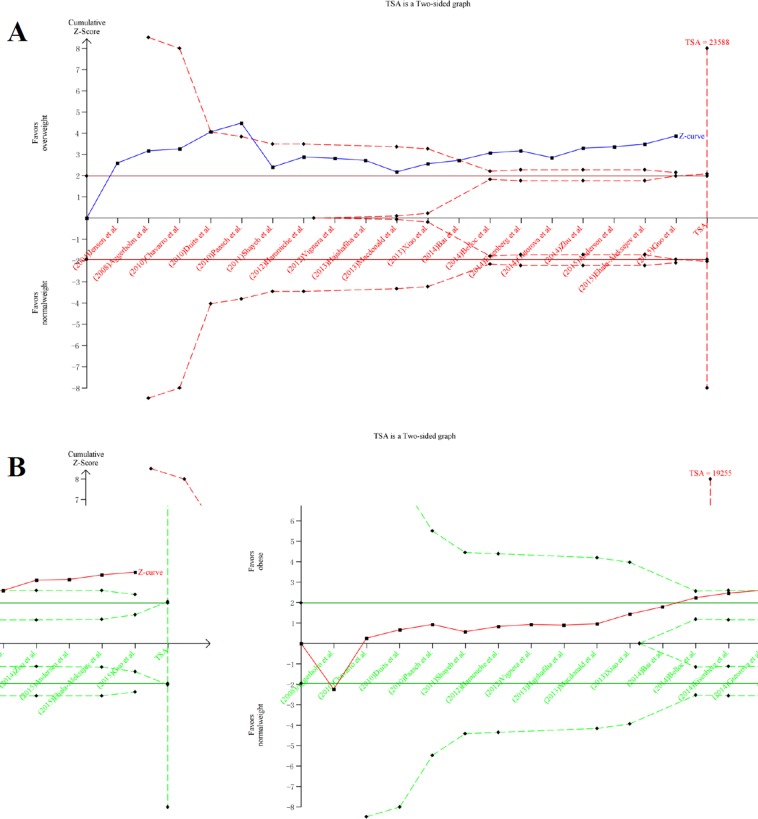
Trial sequential analysis about total sperm count. A. overweight; B. obese

We used trial sequential analyses to calculate the required information size of different sperm parameters in different groups. The results showed that none of the numbers included in the meta-analysis exceed the required information size except semen volume of obese group and some boundary TSA ignoring due to too little information use (Figure 7). Among the positive results in meta-analysis, TSA showed that the cumulative Z-curve (blue line) did cross both the conventional boundary (P = 0.05) and the trial sequential monitoring boundary in total sperm count and semen volume of overweight group and obese group, sperm concentration of obese group, indicating the positive results were confirmed. In contrast, the negative results of TSA of other sperm groups turned out to be true positive.

### Metabolite identification of seminal plasma

The seminal plasma of four groups depending on BMI and sperm parameters (NN: both normal; NA: normal BMI and abnormal sperm parameters; AN: abnormal BMI (obese) and normal sperm parameters; AA: abnormal BMI (obese) and abnormal sperm parameters) were used to perform the metabolomic analysis. (Sperm parameters normal means total sperm count, sperm concentration, semen volume, motility are all above WHO reference lower limits. In contrast, it will be defined as abnormal). We got the preliminary significant changed metabolites profile compared the results of group AA with NN. After eliminated the different ones between group NN and NA, as well as some changed ones between group NN and AN, we caught the final different metabolites list (Table 4), and analyzed metabolic pathways using MetaboAnalyst (http://www.metaboanalyst.ca/). The results showed 5 metabolic pathways were significantly changed, among which the three lowest P values were of arginine and proline metabolism, beta-alanine metabolism and glutathione metabolism (Table 5). Interestingly, the mutual metabolites of these pathways that showed different concentrations of group AA compared with NN were spermidine and spermine. The concentrations of spermidine and spermine were significantly higher in group AA than group NN.

**Table 4:** Statistically significant changed metabolites in seminal plasma

Metabolite	*P* Value	Median case	Median control
Sorbitol	0.004809	1.56884	0.875969
2-Hydroxyglutaric acid	0.005751	0.177469	0.097649
Fumaric acid	0.010213	0.187117	0.241223
Maltose	0.014895	241000	94518
Fructose	0.014956	58.0786	41.5207
Arabionse	0.019451	0.056726	0.025847
Spermine	0.020477	0.017984	0.003356
3a,7a,12a-Trihydroxy-5a-cholestanoic acid	0.023849	0.008571	0.010245
Spermidine	0.023851	0.260766	0.156707
Palmitic acid	0.025077	0.464602	0.375263
Ribose	0.026356	1.18142	0.800664
Cholesterol	0.030544	0.743243	0.412022
Dehydroascorbic Acid	0.030742	0.053261	0.00141
Creatinine	0.035289	15.5022	19.9561
Stearic acid	0.046684	0.217045	0.153005
Aminomalonic acid	0.048856	1.22205	1.98582

**Table 5:** Pathway enrichment of significant changed metabolites

Pathway name	*P* value
Arginine and proline metabolism	0.000395
beta-Alanine metabolism	0.008016
Glutathione metabolism	0.014501
Fatty acid biosynthesis	0.023529
Starch and sucrose metabolism	0.024442
Citrate cycle (TCA cycle)	0.095485
Alanine, aspartate and glutamate metabolism	0.11355
Fatty acid elongation in mitochondria	0.12688
Pentose phosphate pathway	0.14869
Butanoate metabolism	0.18254
Galactose metabolism	0.18669
Nicotinate and nicotinamide metabolism	0.19901
Phenylalanine metabolism	0.20307
Fructose and mannose metabolism	0.21516
Fatty acid metabolism	0.22313
Tyrosine metabolism	0.32016
Amino sugar and nucleotide sugar metabolism	0.36109

## DISCUSSION

In the meta-analysis, we found that high BMI decreased sperm quality such as sperm count, concentration, and semen volume rather than sperm motility (overall or progressive). Meanwhile the trial sequential analyses confirmed the relationship to some extent according to the calculation of the required information size.

Our result for the association between BMI and sperm quality is consistent with some large studies [6, 8, 20], though they showed influence on different sperm parameters. In contrast, our calculation got a different conclusion to the previous relevant meta-analysis published in 2010, which found no relationship between BMI and sperm quality in generally [10, 11]. But it is incredible Sermondade et al found abnormal body weight elevated the risk of oligozoospermia or azoospermia though the whole analysis had a negative result in another meta-analysis published in 2013 [11]. The results of our own unpublished data showed obese men were more like to have total sperm count, sperm concentration and sperm motility below the WHO lower reference limits than normal weight men in infertile part. All of these convince us that there exists a relationship between body weight and sperm quality in general population more than the special crowed. The sample size may be the main issue and the current meta-analysis recruited 25 studies including 26814 individuals, which made up for the deficiency of the previous meta-analysis published in 2013 containing 13077 participants and our own data unpublished including 2106 individuals. In addition, the trial sequence analyses increased the credibility of the results avoiding the false positive in the meta-analysis.

The effect of obesity on the traditional sperm parameters is thought to be multifactorial and the proposed pathophysiological mechanisms underlying these sophisticated relationships have been raised from endocrinology to psychology. First of all, probably of more concern is the changes of hypothalamic-pituitary-gonadal (HPG) axis. Aromatization activity increased by the elevated white adipose tissue of obese men converts testosterone to estrogens, and the enhancive leptin coming from the same source decreases the production of testosterone from Leydig cells [22]. Besides, hypoxemia caused from sleep apnea of obese men is another reason of a decline in morning testosterone concentrations [23]. Decreased testosterone and increased estrogen disrupt the negative feedback loop of the HPG axis, which interfere with the normal progress of spermatogenesis. Secondly, obese men have an increased level of oxidative stress resulting from an increase in seminal macrophage activation, which damage sperm DNA integrity [24]. Thirdly, some researchers proposed another hypothesis that the thermal effect resulting from increased scrotal adiposity could harm sperm cells [25]. Finally, obesity can also be related to erectile dysfunction, and to sexuality in a reverse fashion, due to the psychological impact [26]. Because obesity can be thought as a metabolic disease, we expect new finding from the metabonomics that no one has ever been involved in. For the metabolomic analysis, we found spermidine and spermine had higher concentrations in the group of obese men with abnormal sperm quality. Spermidine and spermine are polyamines, and are essential to male reproductive processes such as testicular development and spermatogenesis. Although evidence for the occurrence of polyamines indicates that it is beneficial and indispensable to spermatogenesis, an excess is detrimental to the progress. Halmekytö M et al studied the effect of superabundant polyamines on spermatogenesis by the transgenic mice [27]. They found the first and second generation male offspring displayed reduced reproductive performance, even infertile or no spermatogenesis. Which implies us it might be the polyamines such as spermidine and spermine that play an important role in the poor sperm quality resulted from obesity. Of course, the precise mechanism needs more relevant deeper researches.

There are several limitations to our study. First, we didn't obtain the original data through contacting the corresponding authors. All the information in the meta-analysis relied on published articles only. It is inevitable that the unity and credibility of data is disturbed to a certain extent because of the variety of BMI categories, statistical approach and outcomes. We used the SMD and converted data format by the accepted formula to reduce the influence as far as possible. Besides, due to the limitation of data sources, the use of different boundaries for normal, overweight and obese in Chinese studies was different from others, which may affect the final results. However, because people of different ethnicities experience various extrinsic factors and they have different genetic sources, the physique is different between Asians and Western populations and we have conducted subgroup analyses based on the ethnicity of participants and different classification standards to reflect the impact of such difference brought about. Second, considering the data processing and statistical aggregation, some relevant articles accessing effect of BMI on sperm quality were excluded even though they maybe provide useful information. In order to verify the reliability of the results, we performed the trial sequence analyses, which confirmed the validity of the present meta-analysis though the sample size didn't meet the requirement. Third, BMI was considered as the unique surrogate of body fat content to evaluate the influence of obesity on sperm quality, and other information such as the relationship between waist circumference [16, 18], or waist-to-hip [18] ratio, or waist-to-height [18] and male infertility was ignored. BMI is flawed and not a perfect index because it has been questioned about its thresholds for overweight and obesity [28], and it does not distinguish fat from fat-free mass [29]. In spite of it, compared with other indicators, BMI is still the most widely used and relatively convenient, which suggesting our findings be more universal and more suitable for the application.

Our results have confirmed that obesity is a pernicious factor of sperm quality. While we look forward to springing out large sample researches to further validate about the relevance between obesity and sperm motility and morphology. According to the conclusion, it would be natural to assume that weight loss will have a beneficial effect on fertility of obese men. However, the truth is still unknown. Several relevant articles have been published about weight loss through dietary, exercise interventions, or bariatric surgery and male fertility. Most reports paid more attention to the effect on the reproductive hormones rather than sperm parameters and the results were ambiguous. Niskanen et al [30] and Kaukua et al [31] found weight loss increased testosterone level, while Leenen et al [32] showed there was no correlation between weight loss and total testosterone level. Likewise, Hakonsen et al [33] found weight loss could improve total sperm count, while Sermondade et al [34] got the opposite conclusion. Further study assessing the effect of weight loss (especially some amusing ways such as changes in meal timing [35]) on the improvement of sperm quality or not is warranted. Besides, the finding of metabolomic analysis suggests us considering from the point of small molecules to explain the phenomenon and apply to clinical therapy. Of course, it also needs more in-depth researches to explore the mechanism and utilize rationally.

Based on this study, sperm quality decreases along with BMI increasing, suggesting obesity may be a detrimental factor of male infertility, though lacking of the raw data may influence the accuracy of the results. Further research is required to identify the role of obesity in male sterility.

## MATERIALS AND METHODS

### Search strategy and selection criteria

We searched relevant studies published until June 2015 about BMI and sperm parameters from PubMed, Embase, Web of Science, and Wanfang database without language restriction. The following combined text and MeSH terms were used: (‘overweight’ OR ‘weight’ OR ‘obesity’ OR ‘BMI’ OR ‘body fat’ OR ‘body weight’ OR ‘body mass index’ OR ‘adiposity’ OR ‘IBW’ OR ‘ideal weight’) AND (‘sperm’ OR ‘semen’ OR ‘spermatozoa’ OR ‘sperm count’ OR ‘sperm concentration’ OR ‘semen quality’ OR ‘semen parameters’ OR ‘sperm quantity’ OR ‘total sperm count’ OR ‘oligozoospermia’ OR ‘azoospermia’ OR ‘semen volume’ OR ‘sperm motility’ OR ‘spermatids’ OR ‘spermatocytes’ OR ‘spermatogonia’). We also searched the references of key articles to identify other relevant studies.

After a primary screen of all titles retrieved from the database, we excluded reviews, studies without BMI or sperm data or studies without results on the relationship between BMI and sperm parameters, experimental or interventional studies, mechanism articles, female researches, studies restricted to men with a particular pathology (such as a varicocele) and studies comparing exposed/non-exposed men. Then the full texts of potentially eligible articles were retrieved, regardless of population size, origin, age or ethnicity. Two review authors independently examined these articles and extracted the data. Any disagreements were resolved by discussion between them. Data retrieved included study characteristics and sperm parameters.

#### Recruitment and screening of personal participants

We recruited 1711 and 653 participants from infertility clinic and maternity clinic respectively to collect semen samples by masturbation and got their basic information (age, weight, height) by self-report. Semen analysis was carried out according to the WHO laboratory manual (2010). After eliminated participants with 1. known azoospermia; 2. other reproductive diseases such as varicocele; 3. BMI < 18.5; 4. data incomplete, 2106 individuals were involved in the analysis finally (including 1461 infertile men and 645 fertile men). We computed the relationship between BMI and sperm parameters on the whole personal participants by regression analysis. Then, we calculated the odds risk (OR) under WHO reference lower limits (5th percentiles) (sperm count < 39 million, concentration < 15 million/ml, volume < 1.5ml, motility < 40% and progressive motility < 32%) in different BMI groups of total participants, fertile men and infertile men respectively.

### Data synthesis and analysis

Meta-analysis was performed using studies that including one or more sperm parameter in the BMI categories as following: mean or median total sperm count, sperm concentration, semen volume, sperm motility, or sperm progressive motility. Data were converted to the form of mean and standard deviation in order to facilitate the calculation. BMI was divided into three levers by WHO criteria: 18.5-24.9 (normal weight), 25.0-29.9 (overweight) and above 30 (obese) kg/m2, except in some Chinese studies that defining 24.0, and 28.0 as boundaries. Participants with a BMI between 18.5-24.9 or 18.5-23.9 were deemed as the reference group. Each sperm parameter of each abnormal weight group was compared to the reference group using SMD, which were calculated by mean, standard deviation and sample size using Stata version 12.0. Then, we performed the dose-response analysis about normal weight, overweight and obese with the data reported in these studies [36]. The values of sperm parameters for normal weight, overweight, obese and the mean BMI of the categories had to be available. We calculated a P value for linearity to test the hypothesis that the coefficient was different from 0.

The I2 statistic was performed to assess possible heterogeneity between studies. We used the random-effect model to evaluate SMD if the P value for heterogeneity was ≤ 0.10 or I2 ≥ 50% that demonstrating a high extent of heterogeneity between studies [37]. Furthermore, a sensitive analysis was conducted to evaluate the effect of a single study on the whole effect. Subgroup analysis was performed to assess the effects between each subgroup based on ethnicity of study population at the same time. We utilized Egger's test and Begg's funnel plot to perform a diagnosis of publication bias. Trial sequential analysis (TSA) was used to minimize the risk of type-I errors of meta-analysis [38]. The required information size was evaluated according to a type-I errors of 5%, a power of 80% and the pooled estimate of all included trials as anticipated variance. After adjusted data of target-metabolites by quality control samples, the Wilcoxon rank-sum test was used for two-group tests of metabolomic analysis of our own study. Stata version 12.0 and TSA software (The Copenhagen Trial Unit, Center for Clinical Intervention Research, Denmark) was used to perform all statistical, and P < 0.05 was defined as statistically significant.

### Metabolomic analysis of seminal plasma

The seminal plasma of infertile participants of our own participants were used to perform the metabolomic analysis according to the previous study [39]. Briefly, seminal plasma was mixed with methionine sulphone (MetSul) and D-camphor-10-sulfonic acid (CSA) that used as internal standards. After removing proteins by filtrating, the mixtures were used to perform CE-TOFMS analysis. Sample was injected with a pressure injection of 50 mbar for 10 s and 25 s for anion and cation mode, respectively. The participants were divided into four groups: NN (n = 55), NA (n = 52), AN (n = 12), and AA (n = 9), while others were kicked out (n = 1333).

## SUPPLEMENTARY MATERIALS TABLES


