# Supplementary material for: The impact of BMI on sperm parameters and the metabolite changes of seminal plasma concomitantly
Source: Oncotarget. 2017 Feb 1;8(30):48619–34. doi: 10.18632/oncotarget.14950 (PMC5564712; doi:10.18632/oncotarget.14950)
Supplement: Supplementary file 1 [file oncotarget-08-48619-s001.pdf]

The impact of BMI on sperm parameters and the metabolite changes of seminal plasma concomitantly

SUPPLEMENTARY MATERIALS

Supplementary Table 1: Characteristics of total participants and sperm parameter outcome in relation to BMI of personal study

	Samples (n)	Overall, n = 2106	BMI 18.5-24.9, n = 1333	BMI 25.0-29.9, n = 670	BMI≥30.0, n = 103	ANOVA *P*-value	Regression analysis: Regression coefficient	Regression analysis: *P*-value
Age (y)	2086	29.79 ± 4.15	29.56 ± 4.10 (n = 1318)	30.32 ± 4.33 (n = 667)	29.18 ± 3.34 (n = 101)	**<0.001**		
BMI (kg/m^2)	2106	24.18 ± 3.32	22.21 ± 1.74	26.84 ± 1.36	32.44 ± 3.14	**<0.001**		
Total sperm count	2106	154.58 ± 182.31	161.46 ± 198.13	142.58 ± 146.20	143.51 ± 177.32	0.075	-0.829	0.488
Sperm concentration	2106	50.18 ± 43.95	51.08 ± 44.53	49.14 ± 43.38	45.26 ± 39.79	0.329	-0.065	0.821
Semen volume	2106	3.10 ± 1.95	3.17 ± 2.17	2.99 ± 1.51	2.97 ± 1.51	0.133	-0.021	0.094
Sperm motility	2106	47.37 ± 23.67	47.04 ± 24.07	48.52 ± 22.65	44.12 ± 24.62	0.149	0.117	0.450
Sperm progressive motility	2106	38.42 ± 20.57	37.82 ± 20.70	39.89 ± 20.07	36.57 ± 21.71	0.067	0.193	0.153

Supplementary Table 2: Odds risks for semen parameter measurements below WHO reference limits in total abnormal weight men of personal study

Sperm parameter	Reference limit (WHO)	Crude odds risk (95% CI)[a]		Adjusted odds risk (95% CI)[b]	
		overweight	obese	overweight	obese
Total sperm count	<39 (×10^6)	0.97 (0.78, 1.21)	**1.58 (1.03, 2.43)**	0.99 (0.80, 1.24)	1.53 (0.99, 2.36)
Sperm concentration	<15 (×10^6/ml)	0.98 (0.78, 1.22)	1.24 (0.79, 1.97)	0.99 (0.79, 1.24)	1.19 (0.75, 1.90)
Semen volume	<1.5 (ml)	1.18 (0.85, 1.64)	1.38 (0.72, 2.67)	1.19 (0.86, 1.66)	1.40 (0.73, 2.71)
Sperm motility	<40%	0.85 (0.70, 1.03)	1.29 (0.86, 1.93)	0.82 (0.68, 1.00)	1.30 (0.86, 1.95)
Sperm progressive motility	<32%	0.84 (0.69, 1.02)	1.28 (0.85, 1.91)	0.82 (0.68, 1.00)	1.29 (0.86, 1.94)

a. Crude odds risk of sperm parameters under WHO reference lower limits (5th percentiles).
b. Adjusted for age.

Supplementary Table 3: Odds risks for semen parameter measurements below WHO reference limits in fertile abnormal weight men of personal study

Sperm parameter	Reference limit (WHO)	Crude odds risk (95% CI)[a]		Adjusted odds risk (95% CI)[b]	
		overweight	obese	overweight	obese
Total sperm count	<39 (×10^6)	1.29 (0.78, 2.14)	1.03 (0.38, 2.77)	1.32 (0.80, 2.19)	0.95 (0.35, 2.57)
Sperm concentration	<15 (×10^6/ml)	1.73 (0.90, 3.33)	1.28 (0.36, 4.56)	1.77 (0.92, 3.42)	1.22 (0.34, 4.36)
Semen volume	<1.5 (ml)	1.09 (0.68, 1.76)	0.81 (0.31, 2.17)	1.12 (0.69, 1.81)	0.74 (0.28, 1.98)
Sperm motility	<40%	1.08 (0.74, 1.58)	0.88 (0.42, 1.85)	1.07 (0.73, 1.57)	1.01 (0.47, 2.14)
Sperm progressive motility	<32%	0.99 (0.68, 1.43)	1.12 (0.56, 2.21)	0.98 (0.67, 1.42)	1.25 (0.62, 2.50)

a. Crude odds risk of sperm parameters under WHO reference lower limits (5th percentiles).
b. Adjusted for age.

Supplementary Table 4: Odds risks for semen parameter measurements below WHO reference limits in infertile abnormal weight men of personal study

Sperm parameter	Reference limit (WHO)	Crude odds risk (95% CI)[a]		Adjusted odds risk (95% CI)[b]	
		**overweight**	**obese**	**overweight**	**obese**
Total sperm count	<39 ($\times 10^6$)	1.08 (0.84, 1.39)	**2.72 (1.58, 4.68)**	1.08 (0.84, 1.39)	**2.69 (1.55, 4.67)**
Sperm concentration	<15 ($\times 10^6$/ml)	1.12 (0.87, 1.44)	**1.93 (1.12, 3.33)**	1.11 (0.86, 1.43)	**1.87 (1.07, 3.27)**
Semen volume	<1.5 (ml)	1.01 (0.63, 1.61)	1.78 (0.73, 4.30)	0.98 (0.61, 1.59)	1.86 (0.76, 4.50)
Sperm motility	<40%	0.90 (0.72, 1.14)	**2.40 (1.36, 4.23)**	0.88 (0.69, 1.11)	**2.47 (1.38, 4.41)**
Sperm progressive motility	<32%	0.92 (0.73, 1.16)	**1.98 (1.14, 3.45)**	0.90 (0.71, 1.13)	**2.03 (1.15, 3.59)**

a. Crude odds risk of sperm parameters under WHO reference lower limits (5th percentiles).

b. Adjusted for age.
